# Curvature‐Induced Kinetic Trapping in Chemical Reactions

**DOI:** 10.1002/jcc.70559

**Published:** 2026-11-09

**Authors:** Bernd Hartke

**Affiliations:** ^1^ Theoretical Chemistry, Institute for Physical Chemistry Kiel University Kiel Germany

**Keywords:** Diels–Alder reaction, kinetic intermediates, kinetic trapping, non‐statistical effects, reaction path curvature

## Abstract

Crossing an activation energy barrier in a chemical reaction needs time because enough kinetic energy needs to redistribute into the barrier‐crossing mode. Kinetic energy redistribution is also needed to get across regions with strong reaction path curvature. This latter fact is frequently overlooked, possibly because barriers and curvature peaks often coincide, and curvature is invisible in the usual, pseudo‐one‐dimensional reaction energy profiles. For a set of related stepwise Diels–Alder reactions, we examine reaction paths, energy profiles and molecular dynamics trajectories, demonstrating three different cases: (1) One with a traditional IUPAC‐conforming potential energy intermediate, in which the surrounding energy barriers coincide with curvature peaks; (2) another case in which the second C–C bond closure is associated with small curvature and no clearly visible energy barrier, accordingly the dynamics towards full ring closure is comparatively fast; and (3) a final case also without an energy barrier for the second C–C bond closure but with a large curvature peak there, leading to exceptionally long kinetic trapping between the two C–C bond formations in the downhill‐energy region. Hence, we advocate for calculating reaction path curvature as simple add‐on to reaction paths, to identify cases warranting a full molecular dynamics study. Besides reaction path curvature, further features are analyzed that contribute to dynamic delays, opening up unconventional reaction control possibilities.

## Graphical abstract

Reaction path curvature helps to detect obstacles to reaction progress in the absence of activation energy barriers.

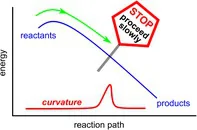

## 1 Introduction

Ideally, chemists would like to do chemical reactions in a highly controlled, single‐molecule fashion, for example, using scanning probe techniques [1]. However, despite published efforts [2], currently it appears impossible to upgrade such approaches to molar amounts of molecules.

Instead, the typical control over thermal (ground‐state) chemical reactions is quite indirect: Reactions are run at such a temperature that only one activation energy barrier can be crossed (or a few of them, with very different heights), leading to the desired products. Inevitably, this barrier can then only be surmounted if a significant amount of the available kinetic (vibrational) energy is concentrated in the degree of freedom that leads to crossing this barrier. Since, this kinetic energy redistribution is quasi‐random, it takes a long time until it actually happens, on the molecular time scale and in molecular dynamics (MD) simulations, making reactions “rare events” in MD. Translating this to features of the potential energy surface (PES), this means that activation energy barriers are associated with curves in the reaction path, which in turn induce kinetic energy redistribution that changes progress along the reaction path from ballistic to long‐time trapping. Accordingly, designing molecules for desired reactions largely focuses on manipulating the electrons towards lowering these PES barriers.

However, barriers and reaction path curves need not coincide exactly. Textbooks remind us of Polanyi's rules [3, 4] for atom+diatom reactions: Translational energy promotes crossing an “early” barrier (barrier before the reaction path curve), vibrational energy promotes crossing a “late” barrier (reaction path curve before the barrier). There have been attempts to generalize Polanyi's rules to polyatomic systems [5] and to reactions on surfaces [6], as well as reports of failures [7] and conceptual extensions [8]. As some of these papers point out, non‐statistical, mode‐selective effects [9, 10, 11, 12] can be seen as a legitimate generalization of Polanyi's rules. Other key concepts of reaction dynamics like the reaction path Hamiltonian (RPH) [13] also feature reaction path curvature as a central element.

Viewing chemical reactions not as sequence of quasi‐static snapshots along a seemingly one‐dimensional reaction path but as full‐dimensional dynamics has a long history. For example, the foundational MD‐papers [14, 15, 16] were already formally reactive. Several general reviews have summarized dynamic effects in reactions [17, 18, 19, 20, 21]. More specifically, dynamic effects in reactions have been documented in a broad range of subfields, for example, in Diels–Alder (DA) reactions [22, 23, 24, 25], re‐arrangements of protonated alcohols [26], reactions involving frustrated Lewis pairs [27], enzyme catalysis [28], single Pd atom catalysis of selective hydrogenation of 1,3‐butadiene [29], Cope rearrangements [30, 31], ring‐editing reactions [32], and homogeneous catalysis by transition‐metal complexes [33]. Dynamics of a DA reaction have also been analyzed via the RPH model [34], even including two explicit solvent molecules, but without discussing delay times (see below) or comparing different DA reactions.

Detection of these phenomena experimentally can be straightforward when the dynamic effects induce one possible product instead of another possible one that was predicted from the quasi‐static viewpoint [26], or when experimental observations like stereochemical or isotopic scrambling can only be explained from the dynamic perspective [32]. Other authors have invoked infrared spectroscopy or the influence of solvent viscosity [27] as possible entry points for experimental detection. For dynamic intermediates with lifetimes shorter than the time needed for significant rotamer exploration to set in, experimental detection is more challenging. In the context of DA and similar multibond reactions this has lead to the concepts of “dynamically concerted” and “dynamically stepwise” [19, 21, 22, 24, 35, 36, 37], for delays between two reaction steps being shorter or longer than 60 fs, respectively. Of course, such timescales can be addressed with femtochemistry techniques, and in fact experimental femtosecond transient soft X‐ray absorption spectroscopy has been used to examine the pericyclic isomerization of 1,3‐cyclohexadiene to 1,3,5‐hexatriene [38].

Of course, all this raises the question if the organic‐chemistry textbook notion of concerted, synchronous reaction steps and the associated orbital symmetry arguments should be abandoned [21, 39, 40]. While Houk's 60 fs criterion discerning between “dynamically concerted” and “dynamically stepwise” certainly is far longer than the idealized zero fs, it should be noted that the 2012 Houk study [22] found delay times of 5 fs or less for symmetrical DA reactions, and even for the most asymmetric case 97% of the trajectories still were “dynamically concerted,” that is, had delay times below 60 fs. Goddard et al. [24] found delay times of up to 240 fs. In a study by Paton et al. [41], average delay times were 365 fs, with four trajectories (3% of the total) showing delay times of 1.8 ps (three times) and 2.6 ps (once). The delay times shown here in the last example are significantly longer and much more frequent, and the case differences are greater, for only subtly different molecules.

In spite of the results briefly reviewed above, there is an obvious deficit amongst chemists to appreciate deviations between quasi‐static reaction paths and energy profiles on the one hand and actual dynamics on the other hand. This deficit has prompted collecting examples of the actual dynamics of molecules in chemical reactions into a database of illustrative MD trajectories [42]. Very recently, a review of dynamic effects in reactions [21] carried the provocative title “Ab initio MD simulations for organic chemists—It is about time!” From the author's perspective, broader adoption of MD studies by non‐specialists is hindered not only by the computational expense but also by a lack of indicators for when to do it. Therefore, we advocate for using reaction path curvature as such an indicator.

Despite very early recognition [13, 43, 44] that curvature peaks are connected to energy redistribution, in particular the link between reaction path curvature and dynamic reaction progress trapping remains underappreciated, as well as the possible non‐coincidence of activation energy barriers with reaction path curvature. For example, the RPH‐based unified reaction valley approach (URVA) by Cremer, Kraka and coworkers [45, 46, 47, 48, 49] uses reaction path curvature as (indirect) key indicator for changes in the electronic system, that is, to separate a full reaction into several different mechanistic steps, without making the above connection to nuclear dynamics. Such a quasi‐static URVA analysis has already been done in great detail for a textbook DA reaction [50], but without pointing out curvature peaks as possible kinetic traps and without a follow‐up of actual dynamics. In prior work [51, 52], we have used the RPH and URVA formalisms to investigate the dynamics of plateau reactions, including analyses of reaction path curvature and couplings. We also examined the dynamics of these reactions in detail, pointing out that transfer of kinetic energy between motion along the reaction path and perpendicular to it occurs in regions of large reaction path curvature (associated with large coupling elements). Admittedly, however, this was a somewhat special class of reactions.

To advocate for reaction path curvature as indicator for kinetic trapping, we have examined DA reactions [53, 54, 55, 56], which is a natural occupation at Kiel University where this reaction was discovered. In addition to the literature cited above, let us re‐emphasize that asynchronous [57] and even stepwise DA reactions do exist [58, 59, 60, 61, 62], not just computationally but also experimentally [63]. In the traditional view, and as pointed out before [64], according to the current IUPAC definition [65], a stepwise reaction should feature in its energy profile two barriers with an intermediate in‐between, giving rise to the possibility of stereochemical scrambling while reaction progress is trapped in the intermediate, where it can start to explore the conformational landscape. In line with the literature cited above, we show that intermediate‐style trapping can occur even if the reaction energy profile does not feature a classic intermediate surrounded by two activation energy barriers. Stepwise DA cases in which the second barrier apparently is absent (one of the cases shown here) have also been reported before, see for example, Figure 2 in [66]. Our examples show that reaction path curvature can be used to pre‐detect the need for MD studies. The MD literature frequently points out [24, 30, 33] that non‐statistical dynamic effects are commonly found in flat PES regions following transition states (TS). In contrast, in the present contribution stepwise DA systems are shown in which the two successive C–C bond formations are associated with only one or even no visible PES barrier and occur along a reaction energy profile region with a straight downward drop in energy by 100 kJ/mol. A seemingly minor molecular modification then determines if the time delay between these two bond formations is on the order of 400 fs or likely longer than 10 ps.

To briefly summarize the state of the art and the main thrust of the present contribution: Various kinetic effects in chemical reactions (including kinetic intermediates and other phenomena) are known to specialists but strongly underappreciated in the broad chemistry literature. Here, we show several DA reactions that appear to be chemically similar but feature dramatically different kinetic behavior, including unusually long delay times for a kinetic intermediate in the last case. This occurs in a PES region with steeply declining energy, which is in contrast to claims in the literature that expect such phenomena in flat PES regions. In the final part of our presentation we point out that such kinetic effects are opportunities to manipulate the reaction dynamics and hence the reaction outcome, beyond the usual thinking about electronic effects; this has been addressed in the literature (e.g., for post‐TS bifurcation) but is not perceived as a general standard concept. Reaction path curvature as cause for kinetic trapping is known in principle but essentially ignored, even in the literature on kinetic intermediates. We argue that this trapping is associated with reaction path curvature, which triggers waiting times for kinetic energy redistribution, and while strong curvature may be associated with energy barriers, there can also be curvature peaks without an apparent energy barrier at the same location. Hence, if curvature is calculated in addition to the usual quasi‐static reaction paths and energy profiles, curvature peaks not associated with energy barriers indicate that a dynamic investigation of the reaction may be warranted.

## 2 Methods

To further lower the barrier towards broader adoption of MD studies, we emphasize strongly that the qualitative approach used in the present work does not require expert tools. Instead, have used unmodified standard methods with default settings wherever possible, even if expert modifications would have been better from a purist view.

Specifically, we have employed the Orca [67, 68] program for all quantum‐chemistry calculations, always using the r
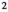
SCAN‐3c method [69] for its superior combination of computational speed and at least semi‐quantitative reliability, combined with the CPCM implicit continuum solvation model [70, 71]. Reaction coordinates and energy profiles were generated with the nudged elastic band (NEB) formalism [72, 73] as implemented in Orca [74], using default settings and 16 images, between pre‐converged local minima for the reactants and for the products, respectively. All MD simulations were also done at the r
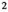
SCAN‐3c‐level with Orca [75], in “direct dynamics” fashion, that is, classical‐mechanical dynamics of the nuclei, with quantum‐mechanical forces re‐calculated on the fly from the electronic system at each time step. These MD calculations employed the same implicit solvent model, a time step of 0.5 fs, and either zero starting velocities with no thermostat or Orca‐generated Boltzmann‐distributed starting velocities corresponding to a selected temperature (ranging from 100 to 400 K), with a Nose–Hoover‐chain thermostat at the same temperature. As a control, several trajectories were also run without thermostat.

As described in the results section, starting geometries for the MD runs were certain NEB images on the reaction path, shortly before or after the cusp of an activation energy barrier. This is somewhat similar to standard literature strategies for calculating “transmission coefficients” to correct transition‐state theory (TST) reaction rates [51, 52, 76, 77]. However, this would require starting directly at the TS, with Boltzmann‐distributed initial velocities only in the forward direction. While such a setup is not difficult to achieve, it is typically not part of the MD capabilities in standard quantum‐chemistry packages like Orca. Also, the aim here is not to calculate transmission coefficients quantitatively but to gain only a qualitative impression of reaction dynamics behavior. Hence, we ensure a bias for the initial movements going in the correct direction by starting at a NEB image not directly at the TS but next to it, and simplify velocity initialization by a general Boltzmann setup in all directions.

Also note the following distinction: The non‐TST effects usually captured by transmission coefficients are recrossing corrections, which clearly are connected to reaction path curvature and hence to kinetic energy redistribution. The effects demonstrated below also violate TST, but they would not be seen as recrossing—mainly because they are not associated with an activation energy barrier and a reactant‐product dividing surface linked to this barrier.

For each case and each temperature (except for 0 K where it makes no sense) we have produced at least 10 trajectories of a simulated length of 10 ps each. These 10 ps are not long enough to cover actual rare events, but they do allow to investigate the short‐time behavior that is of interest here, since, these 10 ps contain many periods of the ring‐closure vibration (cf. Figures 8 and 9 below). Sets of 10 trajectories surely are not large enough to draw quantitative conclusions on small differences but (as shown in the results section) they are sufficient to demonstrate huge qualitative differences in short‐time dynamic behavior.

We also point out strongly that it is *not* important if r
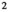
SCAN‐3c (and implicit solvent) describe these reactions sufficiently accurately. Instead, the point is that this level of theory should be good enough that the resulting PESs are realistic, in the sense that they describe actually possible scenarios (for these systems, or for similar systems, or for totally different systems). Additionally, the effects presented here are sufficiently drastic and fundamental that their qualitative features are enough; quantitative accuracy is not important at all.

In the RPH‐based approaches [13], the reaction path for a system of N atoms is the steepest‐descent path (from the TS, in both directions) in mass‐weighted coordinates, that is, a formally one‐dimensional, curved line x(s) in 3N‐dimensional space, with the arc length s measuring progress along this line. The reaction path vector η(s)

(1) $$\eta (s)=\frac{dx(s)}{ds}$$

points along the reaction path, while its curvature vector κ(s) is given by

(2) $$\kappa (s)=\frac{d^{2}x(s)}{ds^{2}}$$

κ(s) is everywhere perpendicular to the reaction path (as illustrated in Scheme 1 of [45]). Its Euclidean norm is the scalar curvature κ(s) used in approximate form below. Further and alternative formulae are provided in [13, 45, 47, 78]. For κ(s)=0, the path is straight; increasing values of κ(s)>0 indicate stronger and stronger bends of the path in 3N‐dimensional space. The usual reaction energy profile plots (cf. Figures 2, 5 and 7 below) show total electronic energy E(s), that is, a one‐dimensional PES‐cut along the reaction path, hiding away its full curvature.

As described in the established literature [13, 45, 48], the RPH‐methodology and its URVA extension then proceed by projecting the total mass‐weighted Hessian onto the molecule‐internal, (3N−7)‐ (or (3N−6))‐dimensional subspace perpendicular to the reaction path and diagonalizing it, obtaining generalized normal modes perpendicular to the path. In harmonic approximation of these modes, this then allows to set up a full Hamiltonian, valid for sufficiently small deviations from the reaction path. Additionally, so‐called Coriolis coupling elements $B_{\mu \nu }(s)$ between the perpendicular modes μ and ν can be derived, as well as curvature coupling elements $B_{\mu s}(s)$ between the perpendicular mode μ and motion along the path.

For the present purposes, a key outcome of the derivations sketched above [13, 45, 48, 51] is that the scalar reaction path curvature κ(s) can also be written as:

(3) $$\kappa (s)={\left(\overset{N_{p}}{\underset{\mu =1}{\sum }}B_{\mu s}^{2}(s)\right)}^{1/2}$$

(for the $N_{p}$ perpendicular modes, disregarding the mode along the reaction path). This clearly indicates that (kinetic) energy transfer between motion along the reaction path and perpendicular to it occurs mainly in regions of strong reaction path curvature. However, according to Equation (2), path curvature κ(s) is defined geometrically, without the need for the full RPH construction. Nevertheless, according to Equation (3), the magnitude of κ(s) still indicates regions of strong/weak (kinetic) energy redistribution.

Reaction path curvature can be calculated with the pURVA program by Kraka et al. [48] that can be obtained from the authors upon request [79]. This requires output from an intrinsic reaction coordinate (IRC) job run with the quantum‐chemistry program Gaussian [80], enhanced with a set of special options. In turn, this delivers the full RPH/URVA analysis sketched above. Alternatively, as just indicated, reaction path curvature κ(s) can also be obtained geometrically, without the need to go through a full RPH/URVA analysis. Hence, as a demonstration and to make reaction path curvature more accessible and compatible with the frequently employed NEB approach (without the need for expert options), we have opted for a different strategy: In a first approximation, we assume that all NEB images are on the reaction path; this certainly is not quite true due to the artificial spring forces employed in the NEB algorithm. To get rid of overall system translation and rotation, all NEB images are aligned to a user‐defined set of four points linked to the molecular system. Each NEB image formally is a point in high‐dimensional nuclear configuration space. Three consecutive NEB images hence, define a plane, containing a circle with these three points on its periphery. Using elementary geometry, the center of this circle (in this plane) can then be constructed easily, and hence also the radius of this circle. Finally, the curvature simply is the inverse of this radius. To ensure that this really works, we have directly compared reaction path curvature calculated with our method and with pURVA, finding good agreement (cf. Supplementary Information, Sections 1.1 and 1.2 with Figures S1 and S2). If desired, individual atomic contributions to the total curvature can be deduced by constructing (by straightforward vectorial projection) the contributions of the coordinates of this atom to the basis vectors spanning the plane of the circle. This alternative approach is approximative and clearly less rigorous and elegant than the RPH/URVA method [78], and hence we are *not*advocating to replace the standard approach with it. However, it serves as an illustration of the key point that path curvature does not require the full RPH/URVA treatment. Hence, path curvature can be added as a simple, independent postprocessing step to any set of molecular structures on a reaction path, generated by any program employing any method without special options, delivering an indicator for locations of strong (kinetic) energy redistribution and for the possible need to complement the quasi‐static reaction path and energy profile view with MD.

No generative artificial intelligence (large language models or other types) was used anywhere in this study, in particular neither in acquiring, analyzing or interpreting the data nor in writing the manuscript.

## 3 Results and Discussion

### 3.1 Regular Stepwise DA Reaction With Two Barriers

In a computational study [81], Domingo et al. have shown that the polar DA reaction between 3‐nitropyridine and Danishefsky's diene does not take place in the concerted synchronous textbook fashion but rather proceeds according to a two‐step mechanism, with a distinct zwitterionic intermediate, as shown in Figure 1. In a recent review article [60] on stepwise DA reactions, this reaction was featured as one of several key examples. We call this “reaction 1” (**Rx1**) in the present study. Note that, Danishefsky's diene has an –${\text{OSiMe}}_{3}$ group, which we have simplified in our dynamics to –${\text{OSiH}}_{3}$, to speed up the calculation of MD trajectories somewhat.

**FIGURE 1:**
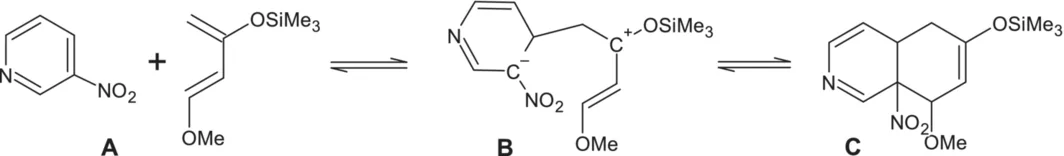
“Reaction 1” (**Rx1**) from [81], see text for details. The labels “A,” “B” and “C” link the molecular structures shown here to Figure 2.

At the r
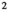
SCAN‐3c level, and with a CPCM solvent continuum model for chloroform (the same implicit solvent as in [81]), we have obtained for **Rx1** the reaction energy profile shown in Figure 2, that is, total electronic energy as a function of progress on the reaction coordinate, from reactants = 0.0 (left in Figure 1) to products = 1.0 (right in Figure 1). This profile features the classic signature of an intermediate, namely two activation energy barriers (corresponding to the two C–C bond formations) with a lower‐energy section in‐between (corresponding to the zwitterionic intermediate). Curvature peaks of the reaction path largely overlap with these two clearly visible activation energy barriers. This interpretation of this profile is corroborated by visual inspection of the NEB images (cf. Supplementary Information, file *rx1_neb.xyz*).

**FIGURE 2:**
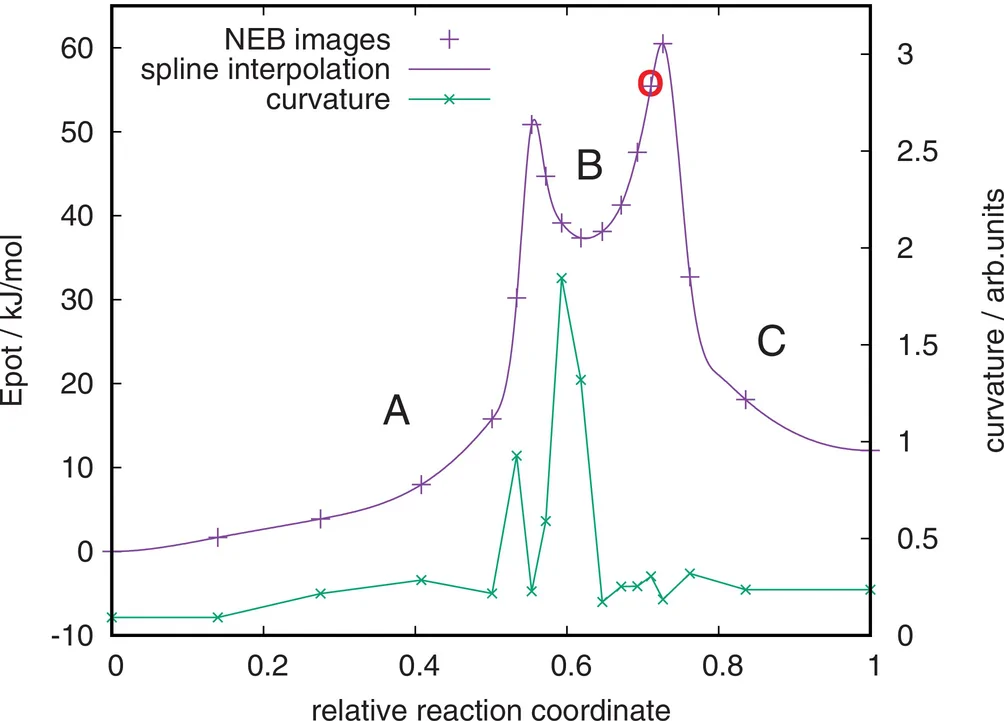
Energy profile of **Rx1** (upper curve, left ordinate), with reaction path curvature (right ordinate), as a function of the relative reaction coordinate (abscissa). Trajectories were started from NEB image number 14, marked by a red circle in this figure. The text labels “A,” “B”, and “C” correspond to the molecular structures shown with the same labels in Figure 1. In the reaction coordinate region to the left of “A” the two reactants re‐orient relative to each other; to the right of “C” the fully formed product relaxes internally.

At NEB image 14, viewed in the “forward” direction (towards the value 1.0 of the relative reaction coordinate), the second C–C bond is about to form. When MD trajectories are started from this image, the expectation is that formation of this second C–C bond will *not* happen but that the system will fall back from this second TS region into the basin of the intermediate. Technically, image 14 has a potential energy slightly above the first TS, so that the ensuing dynamics could lead the system towards complete cycloreversion, that is, the first C–C bond breaks and the separated reactants are obtained (at a value of 0.0 of the relative reaction coordinate). However, breaking the first C–C bond is an activated process, and as indicated by the peaks in reaction path curvature, upon starting at image 14, a substantial amount of kinetic energy first resides in dissociation of the almost‐formed second C–C bond and needs to be redistributed into splitting the first C–C bond. This quasi‐random redistribution takes time, which is the usual scenario in chemical reactions that for this reason are rare events on the MD time scale.

All these standard expectations fit to what is really observed in our MD simulations, started from image 14. At *T* = 0 and *T* = 100 K, the system gets stuck in the intermediate basin and only oscillates around in it, at least within our 10 ps observation window. At 200 K, in 60% of the trajectories, cycloreversion to the reactants actually happens, after delay times ranging from 0.7 to 3.0 ps. This makes sense because at elevated temperatures there is more energy available overall, hence, there is an increased likelihood that sufficient kinetic energy is present in the direction of splitting the first C–C bond, within a sufficiently short time. In one of these 10 trajectories at 200 K, the system escapes at 3.2 ps from the intermediate basin not back to the reactants but towards a side product, depicted in Figure 3. This possibility is well known; quite generally, nitroalkenes can act as diene heteroanalogues in DA reactions [60, 82].

**FIGURE 3:**
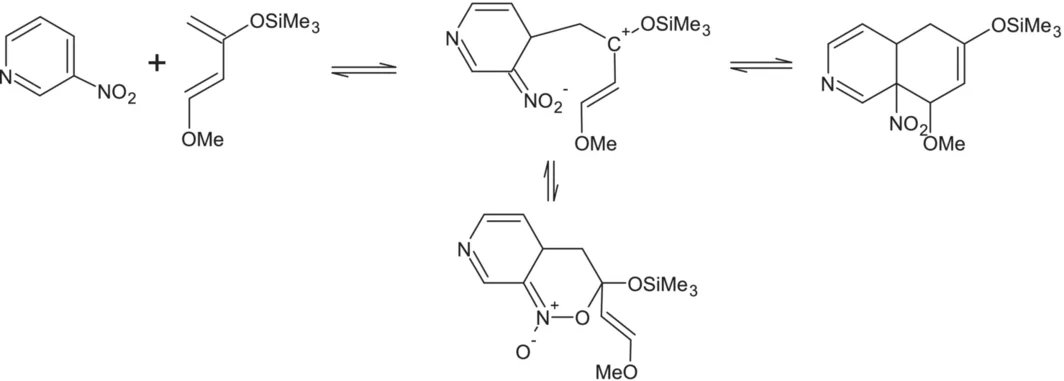
“Reaction 1” (**Rx1**) with its side branch (bottom), see text for details.

At 300 K, the dynamics change again. Still starting from image 14, we now see a quick deviation into the side product. Then, in half of the cases the system gets stuck there, in the other half a return to the intermediate between 1.3 and 8.7 ps is observed, followed quickly by cycloreversion to the reactants. In one of the latter cases, the system escapes from the intermediate not towards the reactants but towards the products (reaction coordinate value 1.0). However, the higher the temperature the smaller importance of PES shapes and reaction path curvatures, which are the focus of this study; hence, we do not discuss further details of this high‐temperature behavior.

Overall, the dynamical behavior in this case is the conventionally expected one, only serving as background baseline for the other cases discussed below.

### 3.2 Pseudo‐Synchronous DA Reaction With a Single Barrier

Linder and Brinck [83] have also investigated stepwise DA reactions computationally. The reactions in this and the following sections are variants of “case 2c” in “scheme 2” of this Linder/Brinck paper. Note that for MD trajectories we have again simplified the –${\text{OSiMe}}_{3}$ group to –${\text{OSiH}}_{3}$ in all cases. We are again using r
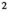
SCAN‐3c and the CPCM implicit continuum solvent model, but as in [83] the solvent now is acetone.

In the present section, we are examining “reaction 2” (**Rx2**), depicted in Figure 4. In contrast to the Linder/Brinck case, our dienophile does not have two CN groups but only one. We treat the case with two CN groups as reactions 3–5 in the next section.

**FIGURE 4:**
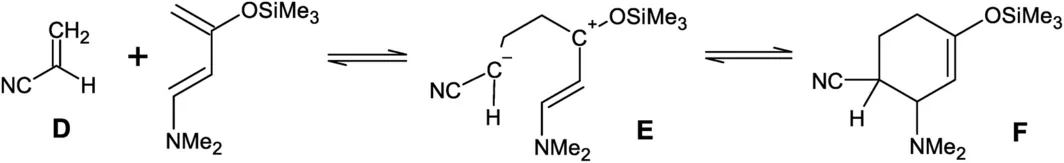
“Reaction 2” (**Rx2**), adapted from [83], see text for details. The labels “D,” “E,” and “F” link the molecular structures shown here to Figure 5.

Figure 5 displays the energy profile and reaction path curvature of **Rx2**. Comparison of this profile with the structures of the NEB images (cf. Supplementary Information, file *rx2_neb.xyz*) reveals that the first C–C bond forms around image 13, at a reaction coordinate value of 0.5, between the labels “D” and “E.” The corresponding barrier with its TS is clearly visible, and it is associated with marked reaction path curvature. The second C–C bond formation happens between images 15 and 16, in the interval 0.6–0.7 of the reaction coordinatem between the labels “E” and “F.” Interestingly, due to the strong decrease in total electronic energy in this region, no clear activation energy barrier is visible and also no significant reaction path curvature.

**FIGURE 5:**
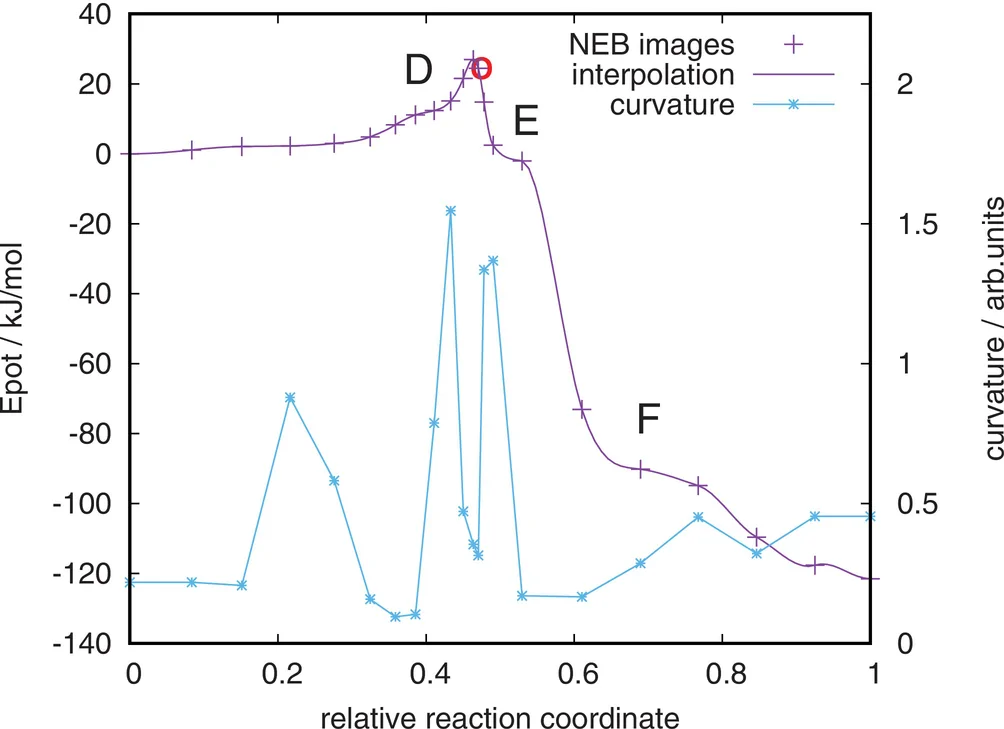
Energy profile of **Rx2** (upper curve, left ordinate), with reaction path curvature (right ordinate), as a function of the relative reaction coordinate (abscissa). Trajectories were started from NEB image number 13, marked by a red circle in this figure. The text labels “D,” “E,” and “F” correspond to the molecular structures shown with the same labels in Figure 4. In the reaction coordinate region to the left of “D” the two reactants re‐orient relative to each other; to the right of “F” the fully formed product relaxes internally.

Quite unsurprisingly, upon starting MD trajectories just after this single visible barrier (image 13, reaction coordinate 0.5), the system always proceeds to the closed product (reaction coordinate 1.0) via closing the second C–C bond, irrespective of the chosen temperature, from 0 to 400 K. The only effect of increasing temperature is that ring closure shifts from 0.4 ps at 0 K to 0.36 ps at 300 K, which can be understood via larger‐amplitude motions at higher temperatures. At 400 K, the kinetic energy available in all degrees of freedom has become so large that the zwitterionic intermediate acquires a visible tendency to open up towards stretched backbone conformers. However, ring closure still always wins, albeit with some time delay (0.7 ps to 1.65 ps).

Note that the curvature peak at reaction coordinate values 0.2–0.3 merely indicates re‐orientation/re‐positioning of the diene and dienophile relative to each other, from their initial reactant‐association minimum to the attack configuration necessary to initiate formation of the first C–C bond.

Overall, despite the absence of a second activation energy barrier, the dynamic behavior in **Rx2** still conforms to traditional expectations: Based on the energy profile, the standard diagnosis would be that there is no intermediate, and hence there can be no stereochemical scrambling. The true reason for this is not that the two C–C bonds are formed at the same time but so fast after each other that exploration of conformational space in the intermediate cannot really happen. Without a detailed femtochemistry analysis, there is no way to experimentally differentiate between these two reasons.

Interestingly, however, the next section shows that a small system change triggers a new, possibly unexpected behavior.

### 3.3 DA Reactions That Get Stuck Without a Barrier

“Reaction 3” (**Rx3**) as depicted in Figure 6 is identical to “case 2c” in “scheme 2” of the Linder/Brinck paper [83] on stepwise DA reactions. The only difference between **Rx2** and **Rx3** is that **Rx2** has one CN group at the dienophile but **Rx3** has two (at the same C‐atom). For our MD simulations, we have again reduced the –${\text{SiOMe}}_{3}$ group to a –${\text{SiOH}}_{3}$ group.

**FIGURE 6:**
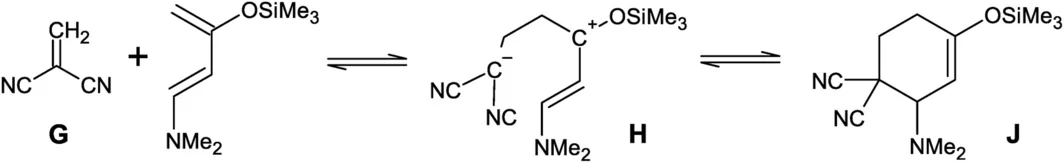
“Reaction 3” (**Rx3**) from [83], see text for details. The labels “G,” “H,” and “J” link the molecular structures shown here to Figure 7.

Figure 7 displays the energy profile and reaction path curvature of **Rx3**. The seemingly broad activation energy barrier in the reaction coordinate region 0.2–0.6 and the first curvature peak around the reaction coordiante value 0.3 again do not really belong to the DA reaction but merely indicate re‐orientation/re‐positioning of the diene and dienophile relative to each other, from their initial reactant‐association minimum to the attack configuration necessary to initiate formation of the first C–C bond. As with the other reactions shown here, this is the outcome of a standard NEB procedure in Orca, deliberately without any attempts to “beautify” it.

**FIGURE 7:**
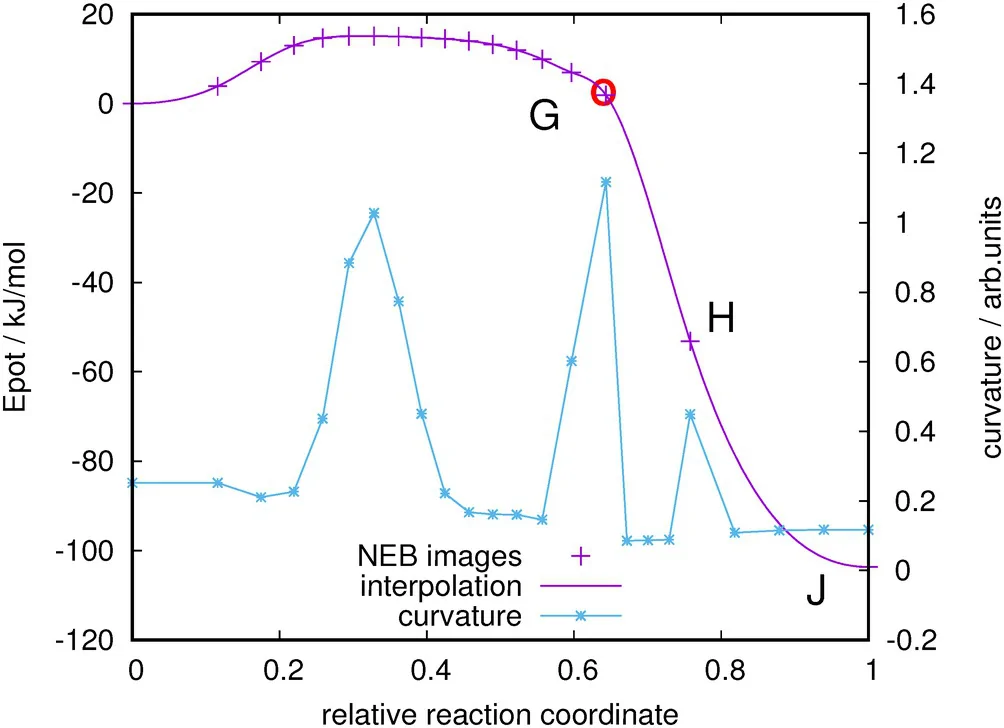
Energy profile of **Rx3** (upper curve, left ordinate), with reaction path curvature (right ordinate), as a function of the relative reaction coordinate (abscissa). Trajectories were started from NEB image number 16, marked by a red circle in this figure. The text labels “G,” “H,” and “J” correspond to the molecular structures shown with the same labels in Figure 6. In the reaction coordinate region to the left of “G” the two reactants re‐orient relative to each other; to the right of “J” the fully formed product relaxes internally.

The first C–C bond forms at image 16 (reaction coordinate value 0.64), which corresponds to the second curvature peak; the third curvature peak at image 17 (reaction coordinate value 0.76) indicates formation of the second C–C bond. This can be confirmed by inspection of the NEB images (cf. Supplementary Information, file *rx3_neb.xyz*) and further illustrated by atomic contributions for these two curvature peaks (cf. Supplementary Information, Section 1.3 with Figure S3). However, neither of these two C‐C bond closures is visible as an activation energy barrier in the energy profile in Figure 7.

Two further variants of **Rx3** are “reaction 4” (**Rx4**), in which the –${\text{NMe}}_{2}$ group is replaced by a –NHMe group, and “reaction 5” (**Rx5**), in which the –NMe2 group is replaced by a –${\text{NH}}_{2}$ group. As shown in Section 2.1, Figure S4, of the Supplementary Information, the energy profiles and reaction path curvatures of these three reactions are very similar.

When starting MD trajectories at image 16 (reaction coordinate value 0.64), the traditional expectation would be that all trajectories should proceed to the ring‐closed product (reaction coordinate value 1.0) without time delays, just as in **Rx2**, because this is a barrier‐less, all‐downhill process. However, the contrast to what actually happens for **Rx3**, **Rx4** and **Rx5** could not be larger: With only a few exceptions (discussed below), the dynamics get stuck between the first and the second C–C bond formation, very much like in **Rx1** with its “obvious intermediate,” and do not escape within our observation window of 10 ps.

From Figures 5 and 7, one might suspect that this strong difference in dynamic behavior could be a trivial result of the energy difference between the starting point and the products being significantly larger for **Rx2** than for **Rx3**. However, in **Rx2** (Figure 5) the product is already fully formed at the label F, which makes the relevant energy differences between the two cases quite similar.

Another possible concern is the thermostat used in the MD trajectories. One can argue that a thermostat is appropriate for simulating a reaction in solution, or one can argue that a thermostat alters the system‐intrinsic dynamics significantly. For this reason, we have repeated six trajectories for **Rx2**without thermostat (but with initial velocities drawn from a Boltzmann ensemble at 100, 200, and 300 K, respectively), and 10 trajectories for **Rx5** without thermostat (again at different initial velocities). The resulting qualitative behavior is almost exactly identical between the thermostatted and not‐thermostatted trajectories. In particular, **Rx2** still always quickly proceeds to full ring closure, on the same timescale as before, while **Rx5** always becomes kinetically trapped between the first and the second C–C bond formation. Two small differences could be observed:

1 Without thermostat, there appears to be a slightly smaller extent of rotamer exploration.
2 While a few cases of full ring closure occur at 300 K for thermostatted dynamics of **Rx5** (as discussed below), none of these were observed without thermostat.

Both of these small effects conform to expectations, however they would need confirmation by larger sets of trajectories. In any case, the strong differences in dynamical behavior do not appear to be influenced by the thermostat at all.

From Figure 7, a possible reason for this dynamic trapping in reactions **Rx3**, **Rx4**, and **Rx5** is the third curvature peak at image 17 (reaction coordinate value 0.76). In other words: Closure of the first C–C bond at image 16 (reaction coordinate value 0.64) requires kinetic energy localized in this region, specifically in this C–C distance, with both C‐atoms moving towards each other. Similarly, closing the second C–C bond requires sufficient kinetic energy there, again with both C‐atoms moving towards each other. But these are two different regions of the molecular system, and two different pairs of C‐atoms. Hence, kinetic energy redistribution needs to occur, from one region to the other, but this is a quasi‐random process involving all degrees of freedom and needs a long time. More generally, reactions are rare events on the MD time scale, not only when activation energy barriers and reaction path curvature peaks coincide but, in cases such as those reported here, apparently also at reaction path curvature peaks that are not accompanied by visible activation energy barriers.

There is some temperature dependence in the observed dynamics. We could not observe any second C–C bond closure for 0 and 100 K, only one for 200 K, and three for 300 K. However, as already observed to a small extent for **Rx2**, higher temperatures induce more exploration of conformational space, and this is more pronounced here. Hence, at higher temperatures, the molecular system can open up into a stretched conformation, in which the second C–C bond closure becomes sterically impossible due to very long distances between the two C‐atoms. Hence, increasing temperatures both enable the second C–C bond formation towards full ring closure but may also prevent it by enabling excursions towards elongated conformations. Apparently, at least for **Rx5**, the net outcome is a window of opportunity towards occasional ring closure around 300 K. Figure 8 gives a summary impression of several characteristic trajectories, by a plot of the C–C distance for the second closure event as a function of propagation time, for five trajectories of **Rx5**: The three full closures at 300 K, the single closure at 200 K, and one of the many non‐closing trajectories, here, one at 300 K that temporarily leaves the ring closure attack conformation towards an elongated conformer, with subsequent return to a different conformer version of the ring closure attack conformation. C–C distances around 2.5 Å correspond to the zwitterionic intermediate, values around 1.6 Å to full ring‐closure, and various stretched conformers of the zwitterionic intermediate exhibit C–C distances in the range 4.5–6.0 Å. Full trajectory files for run1 and run5 of Figure 8 are also supplied (cf. Supplementary Information, files *rx5_run1.xyz* and *rx5_run5.xyz*).

**FIGURE 8:**
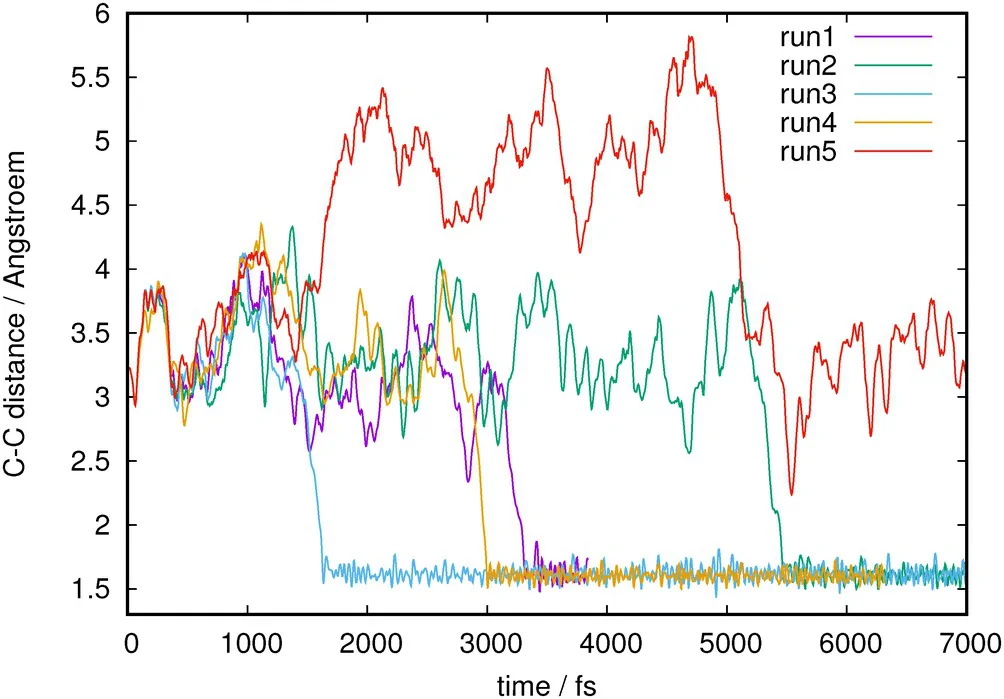
C–C distances (in Ångström) of the second bond formation event, as a function of simulated time (in fs), for **Rx5**. Runs1,2,3 at 300 K, run4 at 200 K, and run5 is a non‐closing but temporarily elongating trajectory at 300 K. Full trajectory files for run1 and run5 are in the Supplementary Information.

Of course, it would be interesting to investigate why we see full closure events only for **Rx5** but not for the very similar **Rx3** and **Rx4**, within our 10 ps observation window. We are not claiming that full ring closure will not happen in the latter two cases but only that it needs more time. Hence, such an investigation would require to extend the MD runs to total simulation times much longer than 10 ps and to enlarge the set of trajectories to hundreds or thousands per case. This is beyond the scope of this initial exploratory study and will be the subject of future work.

Nevertheless, at this early stage we have already collected some observations that allow to formulate reasonable hypotheses why there is fast full ring closure in **Rx2** but not in **Rx3,4,5**. To recall, the key difference is that in the latter cases there are two CN groups at one C‐atom of the dienophile but only one CN group and one H‐atom in **Rx2**. This is a substantial steric difference: The overall extension of the CN group is 2.57 Å (from the dienophile C‐atom to the N‐atom) and it is fairly bulky with its 13 electrons. In contrast, a H‐atom with its single electron is tiny, and its C–H bond distance only is 1.1 Å. Closure of the second C–C bond should ideally proceed from a conformation in which the NC–C–CN axis is perpendicular to the idealized plane of the diene (cf. intermediate in Figure 6). However, inspection of the dynamics of these systems shows significant motion in the twist of the two central dienophile C‐atoms. When the two end C‐atoms happen to be approaching each other towards forming the second C–C bond, significant twist in the NC–C–CN group away from the idealized perpendicular orientation leads to unproductive premature repulsion before ring closure becomes possible. This effect is easily visible in the trajectories accompanying Figure 8. This detrimental effect is reduced if one of the CN groups is replaced by a H‐atom.

In addition to this twist motion allowing full ring closure only around the perpendicular orientation, further dynamic features need to coincide to actually get to closure of the second C–C bond: This C–C distance needs to drop below 2  Å, and simultaneously pyramidalization ($sp^{2}\to sp^{3}$) needs to start at both of these C‐atoms. Figure 9 shows the temporal development of these three degrees of freedom, for two representative trajectories of **Rx2** and **Rx5**, respectively. In both cases, full ring closure finally happens, but at different points in simulated time; the panels show about 1.5 ps time development before this event and a few hundred fs afterwards. Full trajectory files for these two runs are also supplied (cf. Supplementary Information, files *rx5_run1.xyz* and *rx2_fig9.xyz*).

**FIGURE 9:**
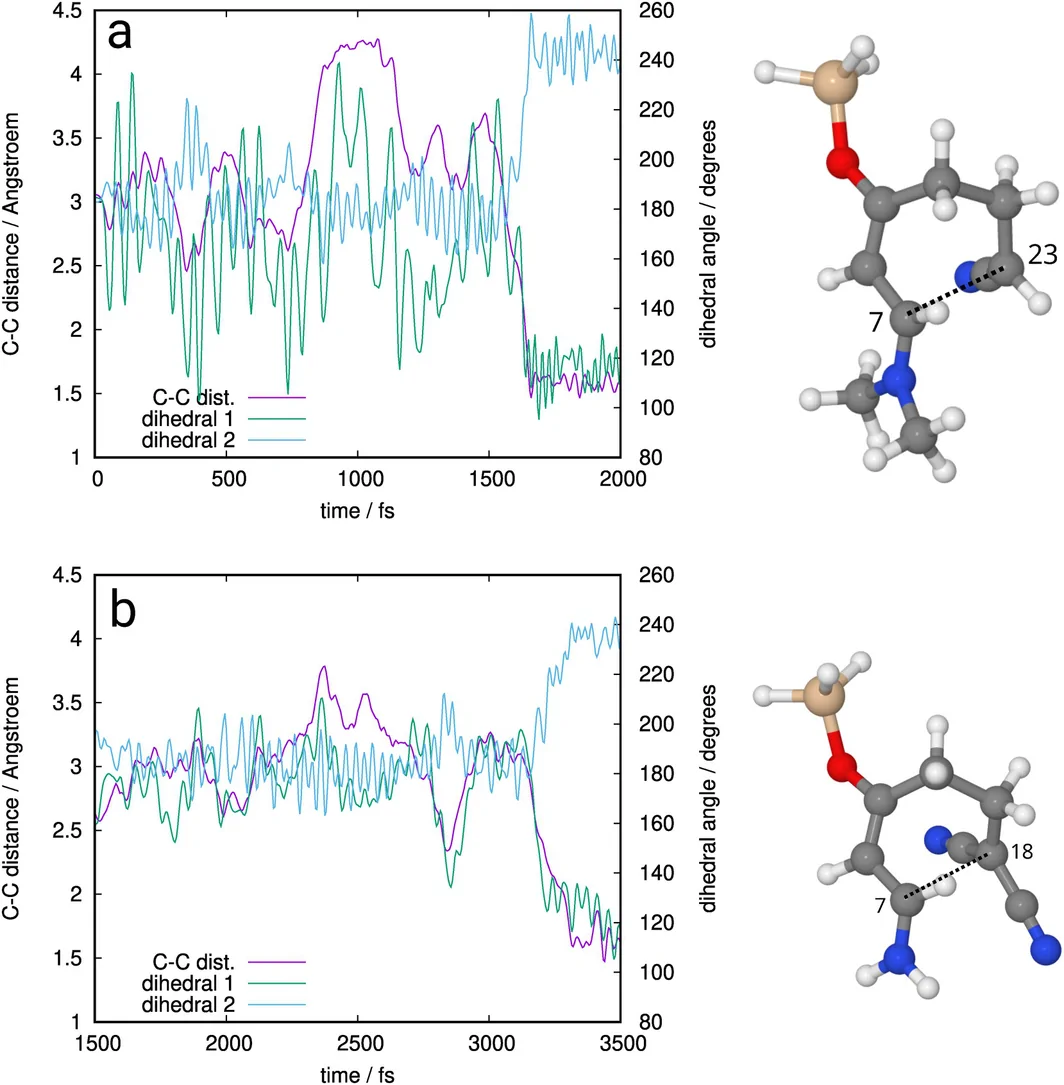
C–C distance (in Ångström, left ordinate) for the final ring closure and pyramidalization (right ordinate) at these two C‐atoms, as a function of simulated time (in fs), for representative trajectories of **Rx2** (panel a) and **Rx5** (panel b). The molecular structure snapshots on the right illustrate the C–C distance with a dotted line; “dihedral 1” pertains to pyramidalization at C‐atom 7 in both cases; “dihedral 2” to C‐atom 23 and 18, respectively. Dihedral angle values around $180^{\circ }$ correspond to $sp^{2}$ hybridization, values around 120° or 240° to $sp^{3}$ hybridization. Full trajectory files are in the Supplementary Information.

Given that the inertia of two CN groups is much greater than that of a CN group and a H‐atom, one may have expected to see larger‐amplitude motion of the C–C distance in **Rx2**, but it is not immediately obvious if this is really the case. Instead, the single big qualitative difference between these two cases is the much larger amplitude of planar‐pyramid deformation at the dienophile C‐atom in **Rx2**. To see this, compare the “dihedral 1” traces in Figure 9a and b, showing the pyramidalization at C‐atoms 23 and 18, respectively, which are identified in the accompanying molecular structure panels. This appears plausible: While the NC–C–CN group has higher inertia and is locally symmetric, the H–C–CN group is strongly asymmetric. Hence, kinetic energy flowing into this group will likely induce torsional‐style motion that is unequal in amplitude and phase between H–C and C–CN, but this is tantamount to pyramidalization.

Additionally, one may suspect electronic differences between **Rx2** on the one hand and **Rx3,4,5**on the other hand, despite their qualitatively similar reaction energy profiles. Again, a full investigation of all directions perpendicular to the reaction coordinate is beyond the scope of the present study. As a first test, however, we have scanned the pyramidalization coordinate at the critical dienophile C‐atom (cf. Section 2.2 of the Supplementary Information, with Figures S5–S7), finding only minor quantitative differences, far smaller than the differences along the reaction coordinate. These differences appear too small to explain the strongly differing dynamic behavior.

As remarked, we have simplified –${\text{OSiMe}}_{3}$ groups to –${\text{OSiH}}_{3}$ groups for our MD calculations, to reduce computational time for them. This affects the energy profiles even less than the changes from –${\text{NMe}}_{3}$ to –NHMe and –${\text{NH}}_{2}$, documented in this section, since the former groups are farther away from the electronics of the ring closure reaction. Since, this change –${\text{OSiMe}}_{3}$ to –${\text{OSiH}}_{3}$ reduces the number of degrees of freedom that are clearly not promoting the reactions towards full ring closure but offers more DOFs into which kinetic energy can redistribute unproductively, going back to the original –${\text{OSiMe}}_{3}$ groups will likely not lessen the phenomenon of these reactions getting stuck between the two C–C bond formations, in terms of kinetic energy redistribution. However, the increased inertia of the –${\text{OSiMe}}_{3}$ groups could conceivably decrease or increase the likelihood of full ring closure. Obviously, also for this reason, further systematic studies with more and longer trajectories are called for, beyond the present first exploratory steps.

## 4 Conclusions

We have taken a small set of similar stepwise DA reactions from the literature and have contrasted their energy profiles with their actual dynamic behavior sampled by sets of short‐time MD runs. These reactions exhibit a broad range of behaviors, some of which may appear counterintuitive from a quasi‐static reaction energy profile view.

One of these reactions has a conventional energy profile with a clearly visible intermediate between two activation energy barriers. As generally expected, the dynamics tend to be trapped between these two barriers for a long time.

Another reaction features two fast consecutive C–C bond formations, but only one of them shows up as a visible barrier in the energy profile. The dynamic behavior is effectively indistinguishable from the textbook synchronous‐concerted one since the time between the two C–C bond formations is too short for conformational exploration. With a time delay of about 0.4 ps between the two C–C bond formations, this is “dynamically stepwise” according to Houk's classification [19, 21, 22]. This stepwise but fast full ring closure is in line with the curvature peak for the second C–C bond formation being fairly small.

Finally, several minor variants of the previous reaction exhibit a long delay in‐between the two C–C bond formations despite the absence of energy barriers or any other signatures for a “true intermediate” in the energy profile. The suspected reason for this strong kinetic trapping is the time delay incurred by kinetic energy redistribution from the region of one C–C bond closure to the separate region of the other, indicated by a strong reaction path curvature peak at the point of the second C–C bond formation.

Redistribution of kinetic energy from one set of DOFs to another should manifest itself as peaks in reaction path curvature, and indeed in these examples this appears to be a helpful diagnostic. Retrospectively, it is quite clear that a pseudo‐one‐dimensional plot of potential energy (total electronic energy) cannot tell the whole story about dynamics on a formally very high‐dimensional PES.

Further analysis has shown that additional effects may contribute to the presence or absence of delays/trappings, including inertia and local symmetry. Generally everything that influences the molecular kinematics and dynamics could come into play here, including different DOF coupling strengths.

With a broader recognition of dynamic effects, the traditional IUPAC definition of what constitutes an intermediate [65] should be updated. Furthermore, it would be of great help if the traditional scheme of calculating reaction path curvature could be implemented in quantum‐chemistry packages, alongside reaction‐path‐finding algorithms. This would provide helpful indications for when to complement the quasi‐static reaction‐path view with actual dynamics studies. For the latter, MD‐capable quantum‐chemistry packages should offer initial MD conditions with user‐defined velocities.

Future work will be devoted to further analyze these examples and also new cases, with a focus on disentangling quasi‐static electronic effects from the various dynamic ones. After decades of emphasis on electronic effects, it will be useful to have dynamic effects as another set of controls to design and guide chemical reactions even better.

## Conflicts of Interest

The author declares no conflicts of interest.

## Supporting information




**Section 1.1:** Curvature comparison between URVA and this work.
**Figure S1:** Energy profile of **Rx2** at the PM3 level (upper curve, left ordinate), with reaction path curvatures (lower curves, right ordinate), as a function of the relative reaction coordinate (abscissa). Curvature has been calculated with our own algorithm (green) and with the URVA formalism (orange).
**Section 1.2:** Sparsely sampled curvature.
**Figure S2:** Reaction path curvature according to our approximate recipe, with the original number of points (as in Figure S1) and with only every seventh point from the same set, bringing the total number of points down to 18.
**Section 1.3:** Atomic curvature contributions.
**Figure S3:** Total reaction path curvature of **Rx3** (as in Figure 7 of the main paper) and two largest atomic contributions to it, clearly differing in their importance in peaks 2 and 3. As shown in the right‐hand panel, atom 23 is the dienophile C‐atom involved in the first C–C bond formation, and atom 17 is a H‐atom in the diene NMe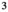 group, peripheral to the second C–C bond formation. The two C–C bonds about to be formed in the DA reaction are indicated by dotted lines.
**Section 2.1:** Energy profiles along reaction path.
**Section 2.2:** Energy profiles for dienophile C‐atom pyramidalization.
**Figure S4:** Reaction energy profiles and reaction path curvatures of **Rx3** (NMe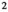), **Rx4** (NHMe) and **Rx5** (NH2).
**Figure S5:** PES cuts along the pyramidalization coordinate of the dienophile C‐atom, comparing **Rx2** (blue, left ordinate) with **Rx3** (green, right ordinate).
**Figure S6:** PES cuts along the pyramidalization coordinate of the dienophile C‐atom, comparing **Rx3** (blue, left ordinate) with **Rx4** (green, right ordinate).
**Figure S7:** PES cuts along the pyramidalization coordinate of the dienophile C‐atom, comparing **Rx3** (blue, left ordinate) with **Rx5** (green, right ordinate).

## Data Availability

The Supporting Information text provides further details on curvature and on PES comparisons. A collection of xyz‐format files in the Supporting Information contain NEB and trajectory data explicitly discussed above, as referenced in the running text. The remaining data generated in this study are available from the author upon reasonable request.
