# Supplementary material for: Curvature‐Induced Kinetic Trapping in Chemical Reactions
Source: J Comput Chem. 2026 Nov 9;47(30):e70559. doi: 10.1002/jcc.70559 (PMC13748696; doi:10.1002/jcc.70559)
Supplement: Supplementary file 1 [file JCC-47-0-s001.zip › New folder/Supplementary_Information.pdf]

*Supplementary Information for:*

# Curvature-induced kinetic trapping in chemical reactions

*Bernd Hartke*
Theoretical Chemistry
Institute for Physical Chemistry
Kiel University
Max-Eyth-Str. 2
24118 Kiel
Germany

hartke@pctc.uni-kiel.de

# 1 Curvature details

## 1.1 Curvature comparison between URVA and this work

To compare our own reaction path curvature calculation (as described in the main text) with the one generated by the original URVA program, we have first reconverged the NEB path for **Rx2** at the semiempirical PM3 level, using Orca, obtaining the energy profile shown in Fig. S1 (the corresponding molecular structures can be found in the supplementary file *rx2_neb_pm3.xyz*). This energy profile is qualitatively similar to the one at the r$^2$SCAN-3c level shown in the main paper. Quantitatively, the activation energy barrier is significantly higher and steeper, which is a typical finding for (traditional) semiempirical methods.

Starting from the NEB image corresponding to the TS, we then started an intrinsic reaction coordinate (IRC) calculation with Gaussian16 [1], in the forward and reverse directions, at the PM3 level and using the special option settings required for URVA. The corresponding output was then post-processed with the pURVA program [2,3], which generates the total scalar curvature in a file *originalkappa.dat*.

An IRC calculation generates significantly more points on the reaction path (here 126) compared to the present NEB settings (here 18). For a fair comparison, we have therefore repeated our own curvature calculation for the larger set of geometries generated by the IRC calculation. For this reason, both curvature plots shown in Fig. S1 are much smoother than those shown in the main paper, which were based on the much sparser NEB images only.

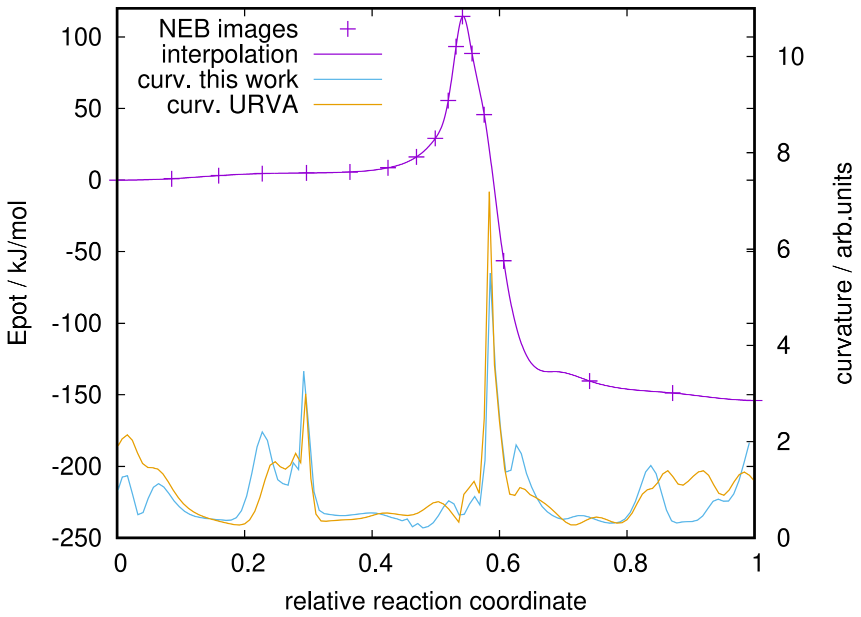


Figure S1: Energy profile of **Rx2** at the PM3 level (upper curve, left ordinate), with reaction path curvatures (lower curves, right ordinate), as a function of the relative reaction coordinate (abscissa). Curvature has been calculated with our own algorithm (green) and with the URVA formalism (orange).

Obviously, the agreement between the two curvature plots is not quantitatively perfect, due to the only approximate removal of total rotation in our method, as remarked in the

main text. However, the qualitative agreement is very good and the semi-quantitative agreement tolerable.

We remark in passing that for the DA reactions studied here, there is only very little difference between calculating total scalar curvature with or without mass-weighting the atomic coordinates. This difference is smaller than that between the two curvatures shown in Fig. S1.

## 1.2 Sparsely sampled curvature

Another possible concern is the finite-difference-like nature of our approximate curvature, when used with only few, widely spaced points. To demonstrate that this does not deteriorate our curvature to being completely useless, we have recalculated it for the same set of PM3 geometries as in Fig. S1, only now skipping successively many points. In Fig. S2 we show the end result of skipping six points (i.e., taking only every seventh point). This brings the total number of points down from 123 (as in Fig. S1) to now only 18, which is comparable to the number of NEB images in the main text.

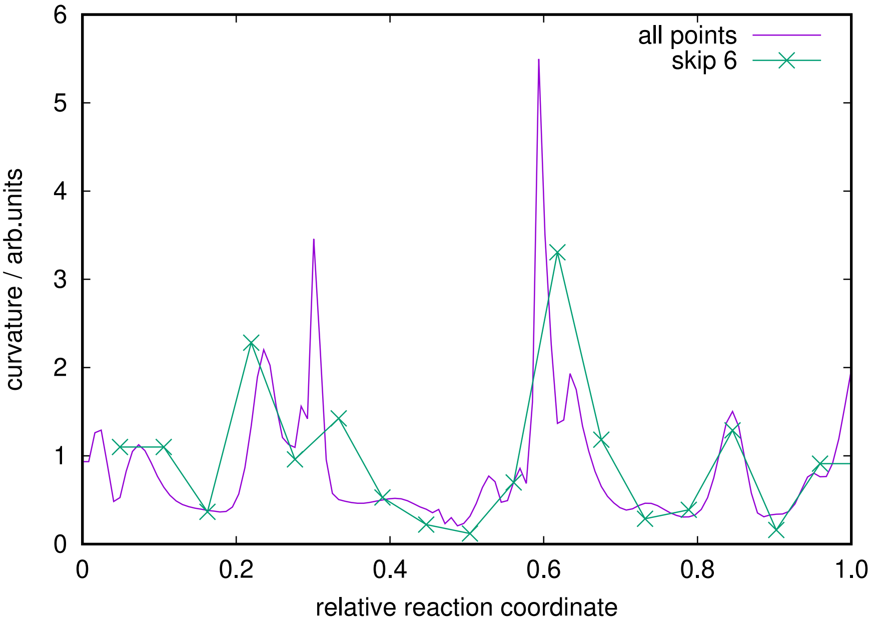


Figure S2: Reaction path curvature according to our approximate recipe, with the original number of points (as in Fig. S1) and with only every seventh point from the same set, bringing the total number of points down to 18.

Obviously, small-scale details like narrow spikes become broadened, but none of them disappear totally. Since in the present study we are aiming at large qualitative effects, we are showing only our own curvatures in low-resolution mode in the main text.

## 1.3 Atomic curvature contributions

Fig. 7 of the main paper displays energy profile and reaction path curvature for **Rx3**. The curvature peak at a relative reaction coordinate value of 0.3 corresponds to re-orientation of the two reactant molecules relative to each other and hence does not

belong to the DA reaction itself. The curvature peak at 0.64 marks the first C-C bond formation, the one at 0.76 the second C-C bond formation.

As mentioned in the methods section of the main paper, atomic contributions to the curvature can be deduced by vectorial projection of center-of-mass coordinates onto the plane constructed to determine the curvature. Fig. S3 shows the two main atomic contributions to the second and third curvature peaks, further confirming that these two peaks arise from motion in two different parts of the molecular system.

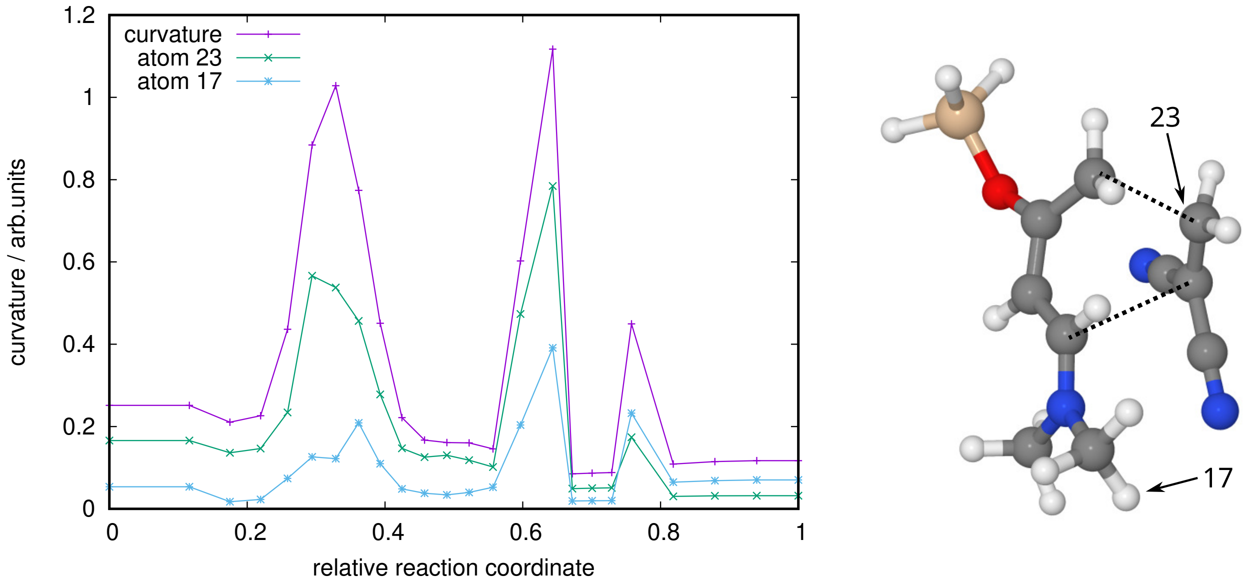


Figure S3: Total reaction path curvature of **Rx3** (as in Fig. 7 of the main paper) and two largest atomic contributions to it, clearly differing in their importance in peaks 2 and 3. As shown in the right-hand panel, atom 23 is the dienophile C-atom involved in the first C-C bond formation, and atom 17 is a H-atom in the diene $NMe_3$ group, peripheral to the second C-C bond formation. The two C-C bonds about to be formed in the DA reaction are indicated by dotted lines.

# 2 Potential energy surface comparisons Rx3, Rx4, Rx5

## 2.1 Energy profiles along reaction path

As indicated in the main text, the reaction energy profiles (cut of the potential energy surface along the reaction coordinate) and reaction path curvatures of the reactions **Rx3**, **Rx4** and **Rx5** are very similar, despite the change of a $NMe_2$ substituent to NHMe and to $NH_2$, respectively, with these substituents being directly attached to the diene (cf. main text). Fig. S4 substantiates this claim, with a direct comparison of these three cases.

## 2.2 Energy profiles for dienophile C-atom pyramidalization

As indicated in the main text, as an entry point to comparing the potential energy surfaces (PES) of the reactions **Rx2**, **Rx3**, **Rx4** and **Rx5** also in directions perpendicular

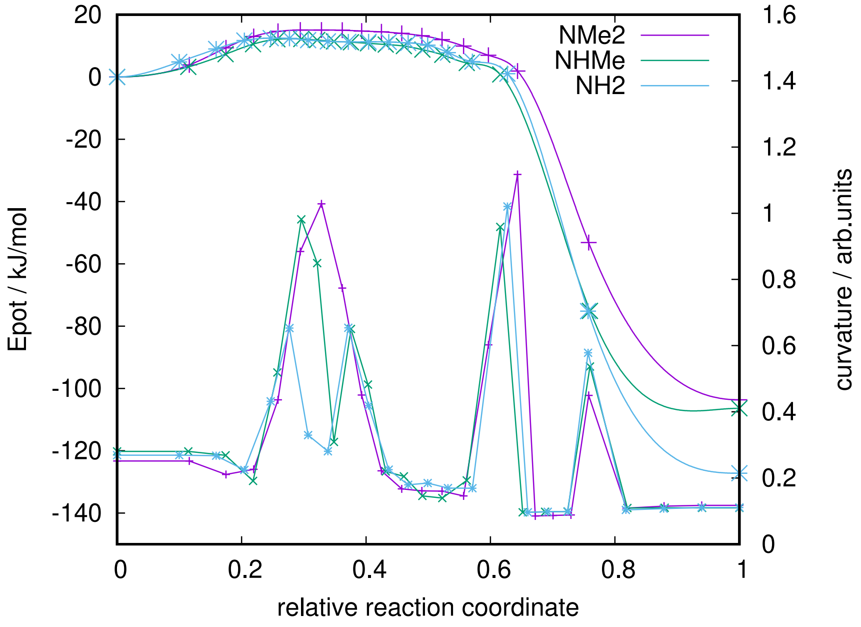


Figure S4: Reaction energy profiles and reaction path curvatures of **Rx3** ($NMe_2$), **Rx4** (NHMe) and **Rx5** ($NH_2$).

to the reaction coordinate, we have prepared and compared PES scans along the pyramidalization of the dienophile C-atom involved in the second C-C bond formation (ring closure), at the point on the reaction path where this second C-C bond starts to form (NEB image 17).

Figs. S5, S6 and S7 compare these cuts in a pairwise manner. Note that despite differing absolute electronic energies, the left and right ordinates always have the same span, enabling direct comparisons also of curve slopes and opening angles.

Obviously, **Rx3**, **Rx4** and **Rx5** are very similar to each other, which is unsurprising, given that they differ only in $NMe_2$, NHMe and $NH_2$ substituents at the diene C-atom *opposite* to the dienophile C-atom in focus here (i.e., its prospective bonding partner).

However, also the cuts for **Rx2** and **Rx2** (cf. S5) are very similar, despite their larger difference: With the C-atom under study here at its center, **Rx2** features a NC−C−CN arrangement, whereas **Rx3** has NC−C−H. Apparently, this does not influence the PES shape in these cuts very much, but as shown and discussed in the main text, the ring closure dynamics differ substantially between these two cases.

Of course, scans of several further coordinates would be of interest, but as indicated in the main text such more detailed investigations are the subject of future work.

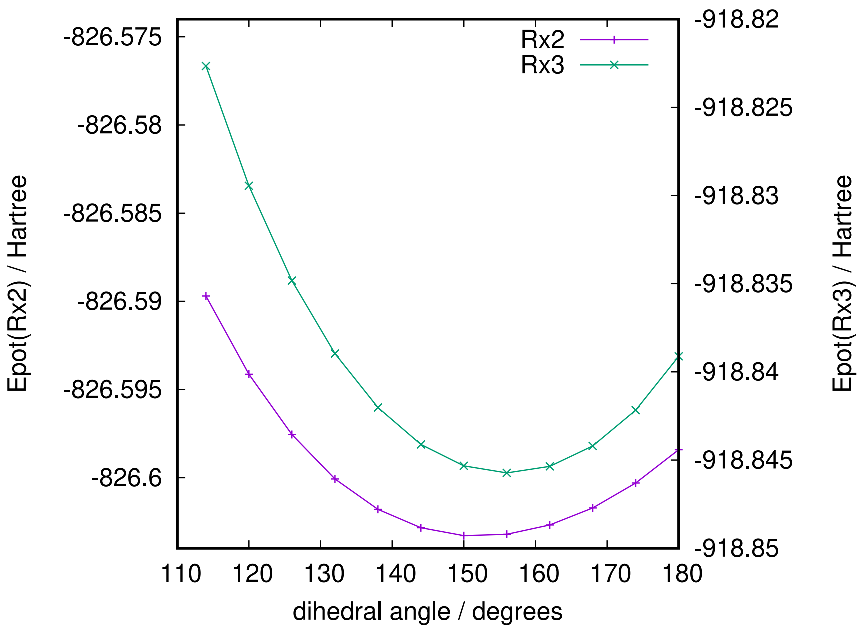


Figure S5: PES cuts along the pyramidalization coordinate of the dienophile C-atom, comparing **Rx2** (blue, left ordinate) with **Rx3** (green, right ordinate)

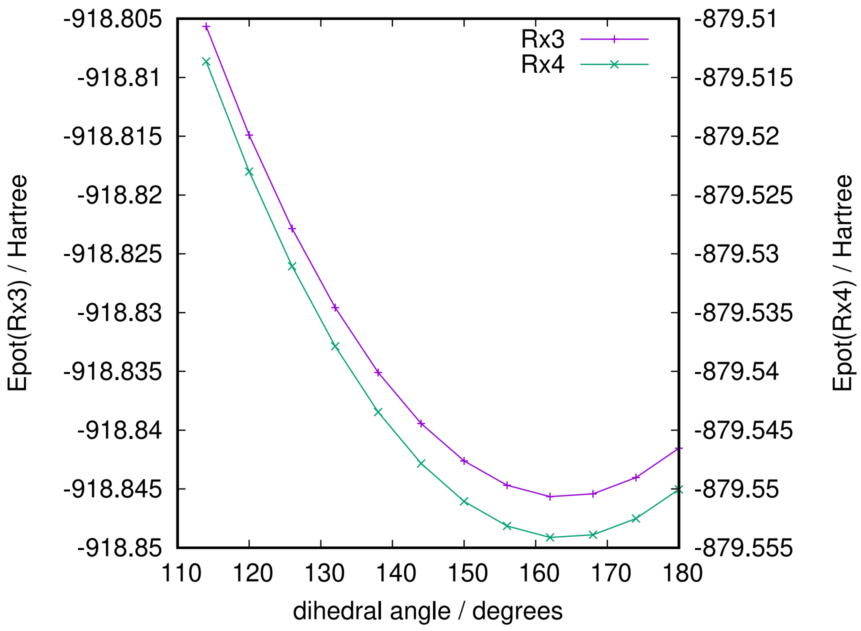


Figure S6: PES cuts along the pyramidalization coordinate of the dienophile C-atom, comparing **Rx3** (blue, left ordinate) with **Rx4** (green, right ordinate)

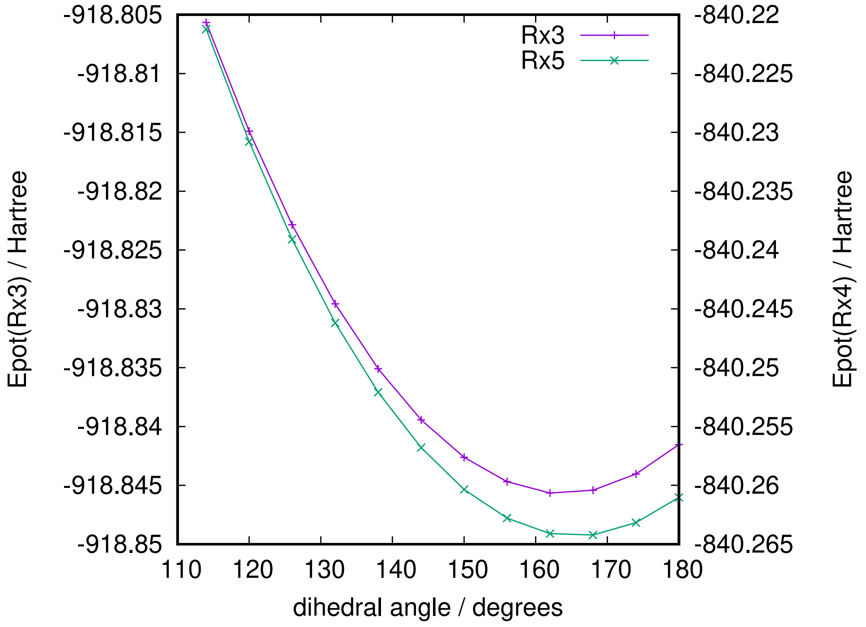


Figure S7: PES cuts along the pyramidalization coordinate of the dienophile C-atom, comparing **Rx3** (blue, left ordinate) with **Rx5** (green, right ordinate)

## 3 List of xyz files provided

**rx1_neb.xyz:** NEB images of **Rx1** at the r$^2$SCAN-3c level, the energies of which are shown in Fig. 2 of the main text.

**rx2_neb.xyz:** NEB images of **Rx2** at the r$^2$SCAN-3c level, the energies of which are shown in Fig. 5 of the main text.

**rx3_neb.xyz:** NEB images of **Rx3** at the r$^2$SCAN-3c level, the energies of which are shown in Fig. 7 of the main text.

**rx5_run1.xyz, rx5_run5.xyz:** Full data (structures as function of time) for trajectories 1 and 5 of **Rx5**, as partially shown in Fig. 8 of the main text.

**rx2_fig9.xyz:** Full data (structures as function of time) for a trajectory of **Rx2**, as shown together with the above `rx5_run1.xyz` in Fig. 9 of the main text.

**rx2_neb_mp3.xyz:** NEB images of **Rx2** at the PM3 level, the energies of which are shown in Fig. S1 of the Supplementary Information.

## References


[1] Gaussian 16, Revision A.03, M. J. Frisch, G. W. Trucks, H. B. Schlegel, G. E. Scuseria, M. A. Robb, J. R. Cheeseman, G. Scalmani, V. Barone, G. A. Petersson, H. Nakatsuji, X. Li, M. Caricato, A. V. Marenich, J. Bloino, B. G. Janesko, R. Gomperts, B. Mennucci, H. P. Hratchian, J. V. Ortiz, A. F. Izmaylov, J. L. Sonnenberg, D. Williams-Young, F. Ding, F. Lipparini, F. Egidi, J. Goings, B. Peng, A. Petrone, T. Henderson, D. Ranasinghe, V. G. Zakrzewski, J. Gao, N. Rega, G. Zheng, W. Liang, M. Hada, M. Ehara, K. Toyota, R. Fukuda, J. Hasegawa,

M. Ishida, T. Nakajima, Y. Honda, O. Kitao, H. Nakai, T. Vreven, K. Throssell, J. A. Montgomery, Jr., J. E. Peralta, F. Ogliaro, M. J. Bearpark, J. J. Heyd, E. N. Brothers, K. N. Kudin, V. N. Staroverov, T. A. Keith, R. Kobayashi, J. Normand, K. Raghavachari, A. P. Rendell, J. C. Burant, S. S. Iyengar, J. Tomasi, M. Cossi, J. M. Millam, M. Klene, C. Adamo, R. Cammi, J. W. Ochterski, R. L. Martin, K. Morokuma, O. Farkas, J. B. Foresman, and D. J. Fox, Gaussian, Inc., Wallingford CT, 2016.

[2] E. Kraka, W. Zou, Y. Tao and M. Freindorf, Catalysts 10 (2020) 691, DOI: 10.3390/catal10060691.

[3] Y. Tao, W. Zou, M. Freindorf, D. Cremer and E. Kraka, Computational and Theoretical Chemistry Group, Southern Methodist University, Dallas, TX, USA, 2018.
